# Baseline 25(OH)D level is a prognostic indicator for bariatric surgery readmission: a matched retrospective cohort study

**DOI:** 10.3389/fnut.2024.1362258

**Published:** 2024-05-13

**Authors:** Yongguang Shang, Mengli Chen, Tianlin Wang, Tianyi Xia

**Affiliations:** ^1^Department of Pharmacy, China-Japan Friendship Hospital, Beijing, China; ^2^Department of Pharmacy, Chinese PLA General Hospital, Beijing, China

**Keywords:** obesity, overweight, vitamin D, bariatric surgery, postoperative complications

## Abstract

**Introduction:**

Managing postsurgical complications is crucial in optimizing the outcomes of bariatric surgery, for which preoperative nutritional assessment is essential. In this study, we aimed to evaluate and validate the efficacy of vitamin D levels as an immunonutritional biomarker for bariatric surgery prognosis.

**Methods:**

This matched retrospective cohort study included adult patients who underwent bariatric surgery at a tertiary medical center in China between July 2021 and June 2022. Patients with insufficient and sufficient 25(OH)D (< 30 ng/mL) were matched in a 1:1 ratio. Follow-up records of readmission at 3 months, 6 months, and 1 year were obtained to identify prognostic indicators.

**Results:**

A matched cohort of 452 patients with a mean age of 37.14 ± 9.25 years and involving 69.47% females was enrolled. Among them, 94.25 and 5.75% underwent sleeve gastrectomy and gastric bypass, respectively. Overall, 25 patients (5.54%) were readmitted during the 1-year follow-up. The prognostic nutritional index and controlling nutritional status scores calculated from inflammatory factors did not efficiently detect malnourishment. A low 25(OH)D level (3.58 [95% CI, 1.16–11.03]) and surgery season in summer or autumn (2.68 [95% CI, 1.05–6.83]) increased the risk of 1-year readmission in both the training and validation cohorts. The area under the receiver operating characteristic curve was 0.747 (95% CI, 0.640–0.855), with a positive clinical benefit in the decision curve analyses. The relationship between 25(OH)D and 6-month readmission was U-shaped.

**Conclusion:**

Serum 25(OH)D levels have prognostic significance in bariatric surgery readmission. Hence, preferable 25(OH)D levels are recommended for patients undergoing bariatric surgery.

## 1 Introduction

The global obesity pandemic is becoming a major public health issue with significant health and economic implications (1). Bariatric surgery is increasingly utilized for patients with obesity and obesity-associated conditions owing to its benefits of significant weight loss, increased life expectancy, and reduced all-cause mortality (2). Weight recovery is the fastest in the first year after the surgery, with a substantial remission of primary comorbidities over the next 5 years (3, 4).

However, although bariatric surgery provides encouraging outcomes, postoperative interventions, such as diet regimens, physical activity, and nutritional management, also play a pivotal role in body weight regulation (5). As obese patients are more likely to have nutritional deficiencies that may emerge, persist, or worsen after surgery (6), nutritional intervention is crucial to achieving favorable surgical outcomes, for which treatment of perioperative malnutrition and prehabilitation are needed. Guidelines commonly recommend nutrient supplementation from the perspective of physiological needs (7, 8), but targeting nutrient deficiencies and the mechanisms by which they affect weight regulation in the long term is of more significance. Therefore, it is recommended that comprehensive and personalized nutritional support be provided to patients with obesity.

Few assessment tools have been validated in patients with obesity. Body mass index (BMI), as a traditional assessment indicator, is unlikely to be a plausible reference for nutritional status because it cannot distinguish between fat and muscle (9). Nutritional risk screening tools independent of BMI, including the Nutrition-Focused Physical Exam, Subjective Global Assessment, and Global Leadership Initiative on Malnutrition, have been proposed along with imaging or anthropometric metrics (10–13); however, the applicability of the above tools still needs to be validated in the obesity cohorts. Therefore, it is imperative to develop a reliable nutritional index to indicate clinical status and outcomes.

Vitamin D plays an important role as a key nutrient in maintaining the balance of calcium and phosphorus metabolism and promoting bone mineralization. It is also involved in cell growth, differentiation, and immunomodulation, participating with other cytokines and growth factors in regulating the local biological behaviors of cells in a network (14). There is a close relationship between vitamin D levels and obesity, with approximately 90% of obese patients experiencing preoperative serum vitamin D deficiency (15). As vitamin D deficiency is well-associated with major comorbidities in obesity, including liver and kidney dysfunction, metabolic and psychiatric disorders, and tumor development (16), effective interventions have been proposed for better prognosis.

Evidence on vitamin D levels and clinical outcomes after bariatric surgery is limited. In this study, we aim to investigate the relationship between baseline serum 25(OH)D levels, a surrogate marker for vitamin D, and postoperative readmission to determine whether the preoperative nutritional status of bariatric patients can be assessed with 25(OH)D and if serum 25(OH)D levels have prognostic implications.

## 2 Material and methods

### 2.1 Study design and population

This retrospective, observational, and matched-cohort study was approved by the institutional review board of the China-Japan Friendship Hospital and was conducted in accordance with the Declaration of Helsinki (2020-132-K85). The need for informed consent was waived owing to the minimal risk from the use of routine clinical records.

Obese patients who underwent bariatric surgery at the China-Japan Friendship Hospital between 1 July 2021, and 30 June 2022, with a 1-year follow-up, were evaluated. The primary endpoint was all-cause readmission at 1 year. The secondary endpoints were the 3- and 6-month readmissions. The inclusion criteria were age > 18 years and a BMI > 25 kg/m^2^ or meeting the criteria for bariatric surgery. The exclusion criteria were as follows: (1) a history of cancer or abnormal tumor indicator; (2) ascites; (3) intensive care unit admission during the perioperative period; and (4) a follow-up time of less than 1 year.

### 2.2 Data collection

Patient data, including clinical diagnosis, medication use, and indications, as well as follow-up information were obtained from the electronic health record database, and the variables were transformed into categorical or ranking ones. The type of bariatric surgery was identified by the surgical procedure. Prognostic nutritional index (PNI) and controlling nutritional status (CONUT) scores were utilized as potential nutritional indicators for comparison. The Charlson Comorbidity Index was calculated to balance the comorbidity risk.

Blood samples were collected for serum 25(OH)D, which was centrifuged for 10 min at 4,000 rpm. 25(OH)D levels were quantitatively determined using an established enzyme immunoassay (EIA) kit (Immunodiagnostic Systems Limited, Boldon, United Kingdom).

### 2.3 Statistical analysis

A retrospective cohort analysis for the enrolled participants was performed. Baseline data were recorded as numbers (percentages) and medians (standard deviations). To identify the biosignificance of nutritional indicators, confounders for 25(OH)D were balanced with preferable propensity score matching (PSM). Patients with 25(OH)D deficiency were matched in a 1:1 ratio by PSM using the nearest-neighbor method to patients in sufficiency.

The datasets were randomly segregated into the training and validation datasets in a 7:3 ratio. Univariate and multivariate analyses were then performed to identify significant indicators of postoperative readmission. For covariate identification, serious models including backward, forward, and stepwise regressions, were compared, and stepwise regression was adopted for the minimal Akaike information criterion value. A fully adjusted logistic regression model employing *a priori* identified confounders was constructed.

A nomogram predicting readmission probabilities after bariatric surgery was plotted using independent prognostic factors in the multivariate regression. Significant variables were represented in the nomogram by a specific value on the horizontal line. The risk score was obtained by adding points for each variable and creating a vertical line, thus predicting the risk of readmission. The discriminative power of the nomogram was validated in both the training and validation datasets. Sensitivity and specificity for the model were evaluated using receiver operating characteristic curves.

The association between 25(OH)D levels and readmission was presented in the restricted cubic spline analysis, with 25(OH)D set as a continuous variable. To visualize and quantify the performance of different nutritional indices, a Sankey diagram was plotted with the three parameters, namely, 25(OH)D level, PNI score, and CONUT score. All statistical analyses were performed using the open-source software R Studio (version 3.5.3, JJ Allaire, USA). *P*-values were two tailed, and *p* < 0.05 was considered significant.

## 3 Results

### 3.1 Patient characteristics

**FIGURE 1:**
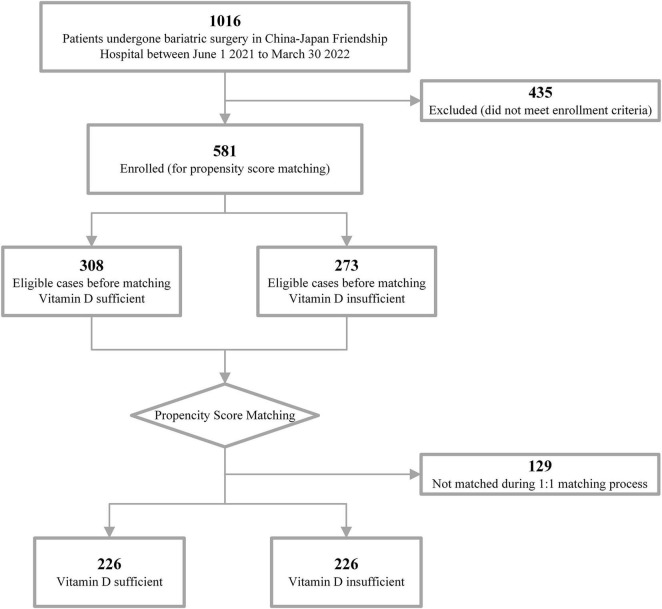
Study flowchart.

A total of 581 patients were included (Figure 1). The median age was 37.25 years, and the median BMI was 38.62. Overall, 67.81% of the patients were female, and comorbidities were identified in 98.11%. For the type of bariatric surgery, gastric bypass was performed in 41 patients and sleeve gastrectomy in 540 patients. With respect to the CONUT score, 216 (37.18%) patients belonged to the intermediate malnutritional risk group and 17 (2.93%) patients to the high malnutritional risk group. The mean 25(OH)D level was 33.47 ng/mL, with a notable insufficiency rate of 46.99% (< 30 ng/mL). Baseline characteristics related to the surgical outcome and 25(OH)D level were significantly different between the sufficient and insufficient groups (Table 1).

**TABLE 1:** Baseline patient characteristics.

		25(OH)D level		
Variable	Total (*n* = 581)	Sufficient (*n* = 308)	Insufficient (*n* = 273)	*p*
Age	37.25 ± 9.68	38.49 ± 9.86	35.84 ± 9.28	0.001
BMI	38.62 ± 8.87	38.21 ± 10.02	39.08 ± 7.35	0.234
25(OH)D ng/mL	33.47 ± 12.26	41.71 ± 10.43	24.17 ± 5.83	0.001
**25(OH)D level, *n* (%)**				0.001
Sufficient	308 (53.01)	308 (100.00)	0 (0.00)	
Insufficient	273 (46.99)	0 (0.00)	273 (100.00)	
Albmin g/L	41.71 ± 10.87	42.20 ± 14.19	41.14 ± 4.90	0.240
Lymphocyte 10^9^/L	1.76 ± 0.74	1.72 ± 0.70	1.82 ± 0.79	0.112
Total cholesterol mmol/L	4.95 ± 2.02	5.04 ± 2.59	4.86 ± 1.07	0.289
Hospitalization time	7.89 ± 3.24	7.85 ± 3.18	7.93 ± 3.30	0.748
**Gender, *n* (%)**				0.022
Female	394 (67.81)	196 (63.64)	198 (72.53)	
Male	187 (32.19)	112 (36.36)	75 (27.47)	
**BMI level, *n* (%)**				0.005
>25	62 (10.67)	37 (12.01)	25 (9.16)	
30–35	151 (25.99)	87 (28.25)	64 (23.44)	
35–40	171 (29.43)	100 (32.47)	71 (26.01)	
>40	197 (33.91)	84 (27.27)	113 (41.39)	
**Operative method, *n* (%)**				0.166
Sleeve gastrectomy	540 (92.94)	282 (91.56)	258 (94.51)	
Gastric bypass	41 (7.06)	26 (8.44)	15 (5.49)	
**Operative season, *n* (%)**				0.001
Summer/autumn	194 (33.39)	123 (39.94)	71 (26.01)	
Winter/spring	387 (66.61)	185 (60.06)	202 (73.99)	
**CONUT, *n* (%)**				0.996
No	348 (59.9)	185 (60.06)	163 (59.71)	
Mild	216 (37.18)	114 (37.01)	102 (37.36)	
Moderate	17 (2.93)	9 (2.92)	8 (2.93)	
**PNI, *n* (%)**				0.118
No	574 (98.8)	303 (98.38)	271 (99.27)	
Moderate	4 (0.69)	4 (1.30)	0 (0.00)	
Severe	3 (0.52)	1 (0.32)	2 (0.73)	
**Charlson Comorbidity Index, *n* (%)**				0.907
1	61 (10.5)	36 (11.69)	25 (9.16)	
2	50 (8.61)	26 (8.44)	24 (8.79)	
3	136 (23.41)	71 (23.05)	65 (23.81)	
4	262 (45.09)	139 (45.13)	123 (45.05)	
5	69 (11.88)	35 (11.36)	34 (12.45)	
6	3 (0.52)	1 (0.32)	2 (0.73)	
**Lifestyle, *n* (%)**				
Smoking	107 (18.42)	58 (18.83)	49 (17.95)	0.784
Alcohol drinking	68 (11.7)	41 (13.31)	27 (9.89)	0.200
Exercise	5 (0.86)	3 (0.97)	2 (0.73)	1
**Comorbidities, *n* (%)**				
Metabolic syndrome	332 (57.14)	177 (57.47)	155 (56.78)	0.867
PCI	4 (0.69)	2 (0.65)	2 (0.73)	1
Hyperlipidemia	297 (51.12)	147 (47.73)	150 (54.95)	0.082
Hypertension	219 (37.69)	130 (42.21)	89 (32.60)	0.017
Apnea	74 (12.74)	41 (13.31)	33 (12.09)	0.659
GRED	455 (78.31)	230 (74.68)	225 (82.42)	0.024
COPD	5 (0.86)	3 (0.97)	2 (0.73)	1
MS	2 (0.34)	0 (0.00)	2 (0.73)	0.220
PCOS	121 (20.83)	53 (17.21)	68 (24.91)	0.023
Infections	5 (0.86)	4 (1.30)	1 (0.37)	0.445
Cardiovascular disease	133 (22.89)	68 (22.08)	65 (23.81)	0.620
Degenerative disease	153 (26.33)	84 (27.27)	69 (25.27)	0.585
Thyroid diseases	54 (9.29)	30 (9.74)	24 (8.79)	0.694
Liver disease	445 (76.59)	226 (73.38)	219 (80.22)	0.052
Renal disease	232 (39.93)	130 (42.21)	102 (37.36)	0.234
**Number of comorbidities, *n* (%)**				0.607
0	11 (1.89)	8 (2.60)	3 (1.10)	
1	16 (2.75)	10 (3.25)	6 (2.20)	
2	23 (3.96)	15 (4.87)	8 (2.93)	
3	47 (8.09)	23 (7.47)	24 (8.79)	
≥4	484 (83.31)	252 (81.82)	232 (84.98)	

### 3.2 Comparison of clinical indicators

**FIGURE 2:**
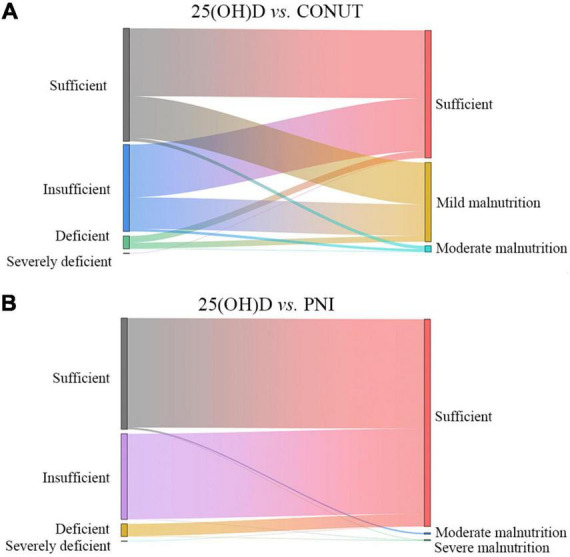
Sankey diagram to visualize and quantify PNI- **(A)** and CONUT- **(B)** defined malnutrition and 25(OH)D levels.

The Sankey diagram for CONUT, PNI, and 25(OH)D of the 581 patients is shown in Figure 2. The rate of 25(OH)D insufficiency was higher than that of malnutrition according to the CONUT score. Only two patients with 25(OH)D deficiency were identified of severe PNI malnutrition. Based on this, it was almost impossible to identify malnutrition through PNI or CONUT at different 25(OH)D levels.

### 3.3 Propensity score matching results

There was no significant difference between the sufficiency and insufficiency groups for 25(OH)D level after PSM. Sensitivity analysis obtained standardized mean difference (SMD) values < 0.1 for all variables. Group comparisons revealed balanced baseline characteristics between the insufficiency and sufficiency groups of 25(OH)D levels. Data distributions before and after matching are presented in Table 2.

**TABLE 2:** Patient characteristics before and after matching for 25(OH)D levels.

Variable	Before PSM						After PSM					
	Total (*n* = 581)	Sufficient (*n* = 308)	Insufficient (*n* = 273)	Statistic	*p*	SMD	Total (*n* = 452)	Sufficient (*n* = 226)	Insufficient (*n* = 226)	Statistic	*p*	SMD
**Age**	37.25 ± 9.68	38.49 ± 9.86	35.84 ± 9.28	*t* = 3.320	<0.001	-0.285	37.14 ± 9.25	37.45 ± 9.29	36.83 ± 9.23	*t* = 0.711	0.477	-0.067
**Gender, *n* (%)**				χ^2^ = 5.242	0.022					χ^2^ = 0.376	0.540	
Female	394 (67.81)	196 (63.64)	198 (72.53)			0.199	314 (69.47)	160 (70.80)	154 (68.14)			-0.057
Male	187 (32.19)	112 (36.36)	75 (27.47)			-0.199	138 (30.53)	66 (29.20)	72 (31.86)			0.057
**Surgery type, *n* (%)**				χ^2^ = 1.916	0.166					χ^2^ = 0.653	0.419	
Sleeve gastrectomy	540 (92.94)	282 (91.56)	258 (94.51)			0.129	426 (94.25)	215 (95.13)	211 (93.36)			-0.071
Gastric bypass	41 (7.06)	26 (8.44)	15 (5.49)			-0.129	26 (5.75)	11 (4.87)	15 (6.64)			0.071
**Surgery season, *n* (%)**				χ^2^ = 12.622	<0.001					χ^2^ = 0.166	0.684	
Summer/autumn	194 (33.39)	123 (39.94)	71 (26.01)			-0.317	140 (30.97)	72 (31.86)	68 (30.09)			-0.039
Winter/spring	387 (66.61)	185 (60.06)	202 (73.99)			0.317	312 (69.03)	154 (68.14)	158 (69.91)			0.039
**T2DM, *n* (%)**				χ^2^ = 0.647	0.421					χ^2^ = 0.010	0.922	
No	203 (34.94)	103 (33.44)	100 (36.63)			0.066	161 (35.62)	80 (35.40)	81 (35.84)			0.009
Yes	378 (65.06)	205 (66.56)	173 (63.37)			-0.066	291 (64.38)	146 (64.60)	145 (64.16)			-0.009
**Charlson Comorbidity Index, *n* (%)**				χ^2^ = 1.550	0.907					χ^2^ = 0.813	0.976	
1	61 (10.5)	36 (11.69)	25 (9.16)			-0.088	43 (9.51)	22 (9.73)	21 (9.29)			-0.015
2	50 (8.61)	26 (8.44)	24 (8.79)			0.012	40 (8.85)	18 (7.96)	22 (9.73)			0.06
3	136 (23.41)	71 (23.05)	65 (23.81)			0.018	110 (24.34)	56 (24.78)	54 (23.89)			-0.021
4	262 (45.09)	139 (45.13)	123 (45.05)			-0.002	198 (43.81)	100 (44.25)	98 (43.36)			-0.018
5	69 (11.88)	35 (11.36)	34 (12.45)			0.033	58 (12.83)	29 (12.83)	29 (12.83)			0
6	3 (0.52)	1 (0.32)	2 (0.73)			0.048	3 (0.66)	1 (0.44)	2 (0.88)			0.047
**BMI, *n* (%)**				χ^2^ = 12.952	0.005					χ^2^ = 3.322	0.345	
>25	62 (10.67)	37 (12.01)	25 (9.16)			-0.099	42 (9.29)	17 (7.52)	25 (11.06)			0.113
30–35	151 (25.99)	87 (28.25)	64 (23.44)			-0.113	123 (27.21)	64 (28.32)	59 (26.11)			-0.05
35–40	171 (29.43)	100 (32.47)	71 (26.01)			-0.147	136 (30.09)	74 (32.74)	62 (27.43)			-0.119
>40	197 (33.91)	84 (27.27)	113 (41.39)			0.287	151 (33.41)	71 (31.42)	80 (35.40)			0.083
**Unhealthy lifestyle, *n* (%)**				χ^2^ = 0.001	0.977					χ^2^ = 0.013	0.91	
No	453 (77.97)	240 (77.92)	213 (78.02)			0.002	351 (77.65)	175 (77.43)	176 (77.88)			0.011
Yes	128 (22.03)	68 (22.08)	60 (21.98)			-0.002	101 (22.35)	51 (22.57)	50 (22.12)			-0.011

### 3.4 Prognostic indicators

Among the 452 matched patients, 316 and 136 patients were included in the training and validation cohorts for prognostic, among which 25 patients were readmitted during follow-up. The CONUT and PNI data was further crossed with 25(OH)D levels for readmitted patients in a Sankey plot (Supplementary Figure 1). Univariate logistic analysis showed that the 25(OH)D level was negatively correlated with 6-month and 1-year readmission (no available data at 3 months). The odds ratio (OR) and 95% confidence interval (Cl) were 5.18 (95% CI, 1.13–23.78) and 3.58 (95% CI, 1.16–11.03), respectively. In addition, surgery season in summer or autumn was also associated with a higher incidence of readmission with OR values at 8.28 (95% CI, 2.22–30.79) and 2.68 (95% CI, 1.05–6.83) (Table 3). Similar results were obtained in the multivariate analysis, denoting 25(OH)D level and surgery season as independent prognostic indicators (Table 3).

**TABLE 3:** Association of potential covariates with 1-year readmission in the fully adjusted univariate and multivariate logistic models.

	Univariate analysis		Multivariate analysis	
**Variables**	**OR (95%CI)**	***p***	**OR (95%CI)**	***p***
**Age**				
	0.97 (0.92–1.03)	0.351		
**25(OH)D**				
Sufficient	Ref.		Ref.	
Insufficient	3.58 (1.16–11.03)	0.027	3.56 (1.13–11.28)	0.031
**T2DM**				
Yes	Ref.			
No	1.12 (0.43–2.92)	0.824		
**Gender**				
Female	Ref.			
Male	0.43 (0.12–1.52)	0.190		
**Surgery type**				
Sleeve gastrectomy	Ref.			
Gastric bypass	0.92 (0.12–7.27)	0.933		
**2009 CKD-EPI eGFR (mL/min/1.73 m^2^)**				
≥ 90	Ref.			
< 90	1.76 (0.56–5.59)	0.335		
**Surgery season**				
Winter/spring	Ref.		Ref.	
Summer/autumn	2.68 (1.05–6.83)	0.039	3.48 (1.31–9.19)	0.012
**Charlson Comorbidity Index**				
≥4	Ref.			
< 4	0.90 (0.35–2.29)	0.820		
**BMI**				
≤ 40	Ref.			
> 40	1.88 (0.74–4.79)	0.183		
**Unhealthy lifestyle**				
No	Ref.			
Yes	0.93 (0.30–2.91)	0.905		
**CONUT malnutrition**				
No	*Ref.*			
Yes	0.59 (0.21–1.68)	0.323		
**PNI malnutrition**				
No	Ref.			
Yes	0.00 (0.00–Inf)	0.991		
**Perioperative comorbidity**				
No	Ref.			
Yes	0.00 (0.00–Inf)	0.992		
**Postoperative vitamin D supplement**				
Yes	Ref.			
No	0.66 (0.23–1.89)	0.442		

### 3.5 Sensitivity analysis for 25(OH)D

**FIGURE 3:**
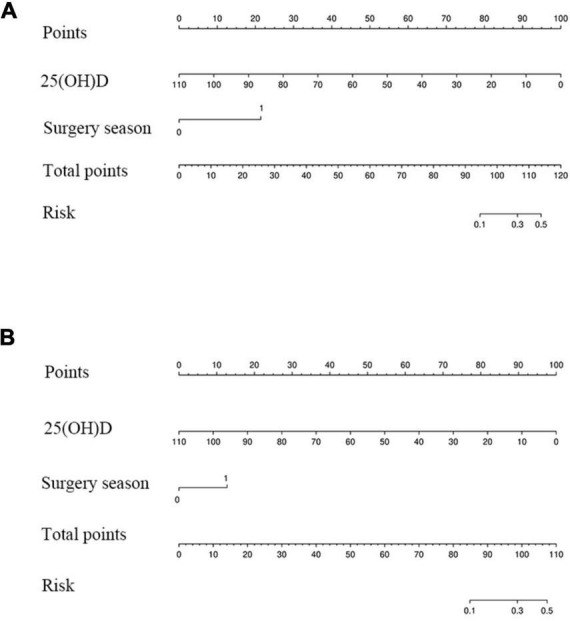
Nomogram for predicting 6-month **(A)** and 1-year **(B)** readmission.

**FIGURE 4:**
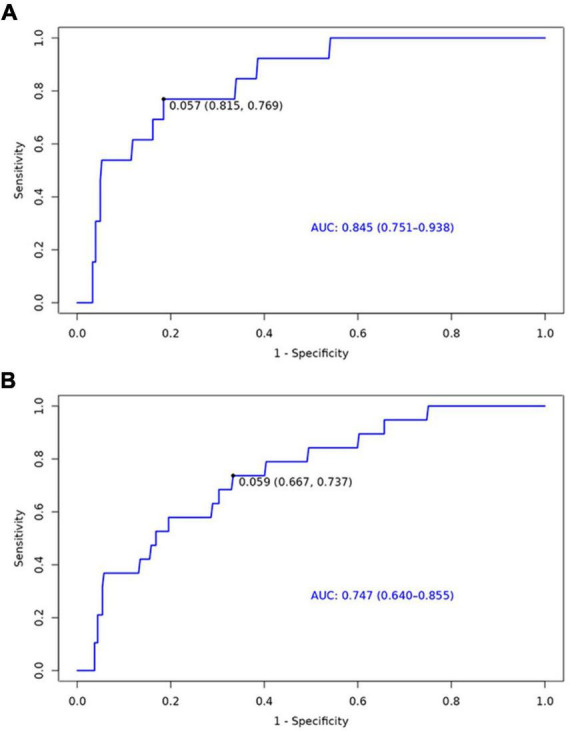
Receiver operating characteristic (ROC) curves for the ability of the nomogram to predict 6-month **(A)** and 1-year **(B)** readmission.

**FIGURE 5:**
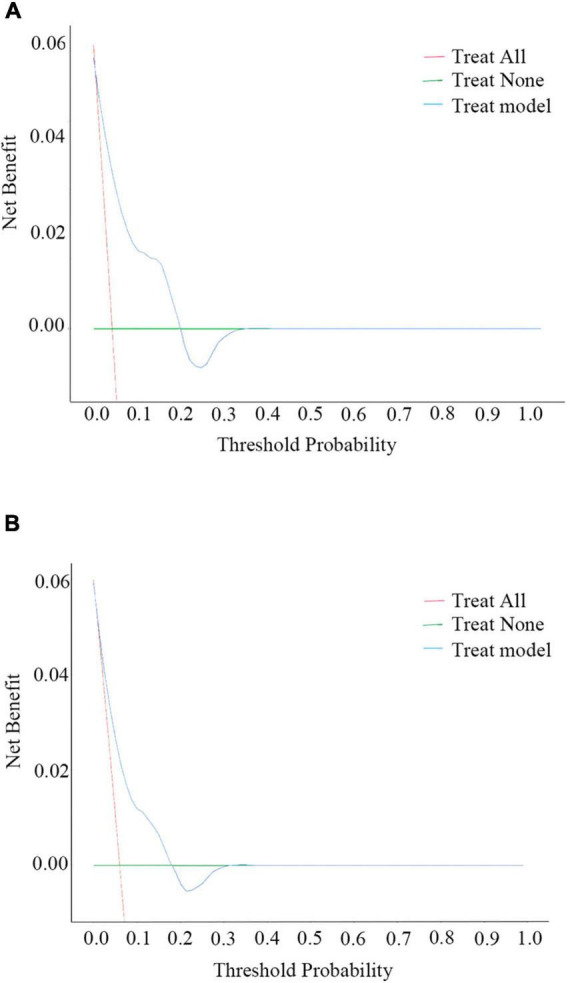
Decision curve analysis (DCA) plots for the ability of the nomogram to predict 6-month **(A)** and 1-year **(B)** readmission.

The nomogram for predicting the 6-month and 1-year readmission probabilities after bariatric surgery is shown in Figure 3. The AUC values for predicting 6-month and 1-year readmissions were 0.845 (95% CI, 0.751–0.938) and 0.747 (95% CI, 0.640–0.855), respectively (Figure 4). In the decision curve analyses depicting the net clinical benefit of the nomogram, the clinical interventions guided by our nomogram achieved positive clinical benefits over guidance with other scoring systems in both the training and validation sets (Figure 5).

**FIGURE 6:**
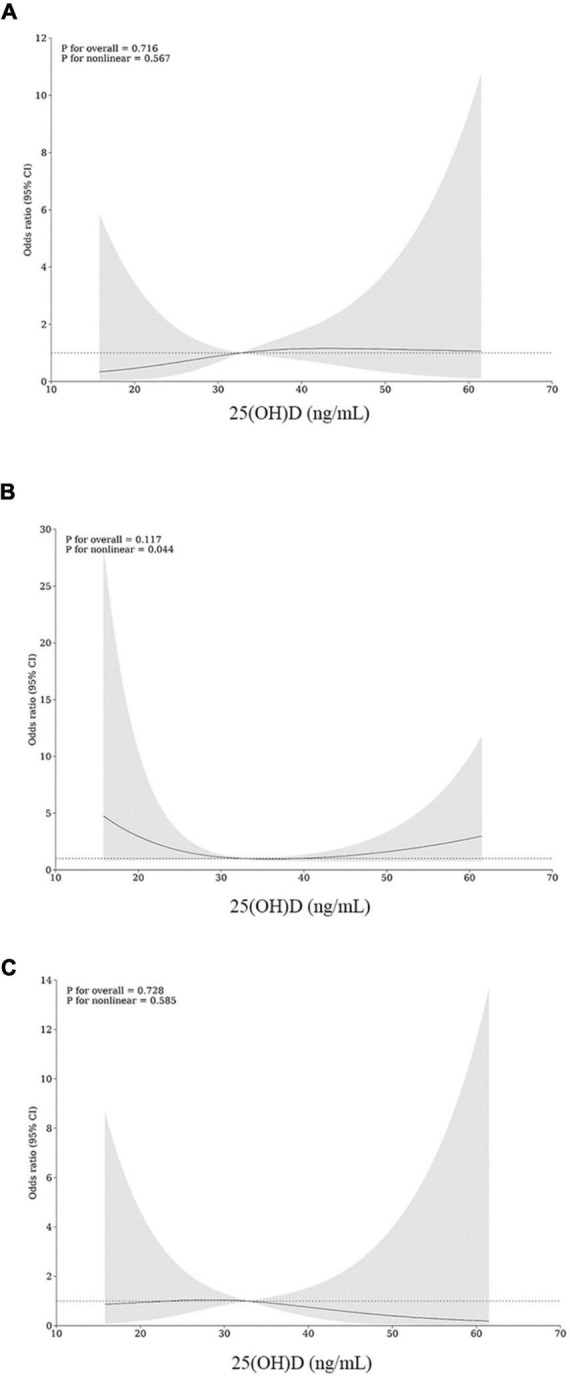
Restricted cubic spline (RCS) for non-linear associations of 25(OH)D levels with and 3-month **(A)**, 6-month **(B)**, and 1-year **(C)** readmission.

The restricted cubic spline plots for the possible non-linear relationships between 25(OH)D levels and readmission are shown in Figure 6. The nodes at 3 were selected as the lowest values for the Akaike information criterion. In the secondary outcomes, there was a U-shaped relation between 25(OH)D levels and 6-month readmission (*p* for overall = 0.117, *p* for non-linear = 0.044).

## 4 Discussion

Bariatric surgery is considered a proactive treatment modality at the time of weight plateau, providing reliable, durable, and greater total weight loss. However, evidence for prognostic estimation of potential postoperative complications is scarce, especially for those who undergo gastric bypass. This study found an increased risk of comorbidities in patients with low baseline vitamin D levels who underwent bariatric surgery in the summer and autumn, adding up instrumental evidence that 25(OH)D level might be indicative of nutritional status and clinical outcomes in bariatric surgery.

The clinical diagnosis of obesity is more challenging when combined with malnutrition. Imaging techniques, including bedside ultrasound and tomography and magnetic resonance imaging, are not commonly employed for assessment as they require skilled interpretation of clinical inferences. For anthropometric indicators, body composition analysis and bioimpedance techniques are not easily accessible. Furthermore, although novel indices, such as A body shape index, body roundness index, and lipid accumulation product, provide a comprehensive and accurate reflection of body fat distribution and muscle mass (17, 18), they have not been widely applied owing to potential racial heterogeneity or generalizability.

Obese patients usually present with altered inflammatory responses and energy expenditure, thus requiring personalized nutritional considerations. Therefore, we proposed that an immune-related nutritional assessment could provide a new point for nutritional support in obese patients (19, 20). To investigate the pathological mechanisms underlying obesity, the PNI and CONUT scores were included in this study for potential assessment in both malnutrition and systemic inflammation in patients with obesity. However, the results indicated that both were ineffective in identifying malnutrition for all the enrolled or readmitted patients despite CONUT being able to identify nutritional risk at a higher level. Furthermore, no statistical significance was found between the two indicators upon readmission. Although PNI and CONUT were proposed in previous studies for nutritional assessment (21–23), the differences in target populations, types of surgeries, and focus on readmission might have caused differences in these study findings. Therefore, there is a need for more sensitive and specific indicators in the assessment of nutritional status and prognosis in obese patients.

The baseline variables utilized for propensity score matching included demographic, lifestyle, and clinical covariates associated with vitamin D levels. Patients with chronic severe vitamin D deficiency endure reduced intestinal absorption of calcium and phosphorus, leading to comorbidities of secondary hyperparathyroidism and osteoporosis (24, 25). As calcium was not assessed in the preoperative period in bariatric patients in this study, to diminish potential bias in data mining, comorbidities of hyperparathyroidism and osteoporosis were recorded and characterized as renal/degenerative diseases for PSM, and eGFR (2009 CKD-EPI estimated glomerular filtration rate) was enrolled as an adjusting variable for prognostic indicator identification.

This study preliminarily revealed vitamin D deficiency as a risk factor for postoperative readmission in bariatric patients instead of the well-established nutritional indicators of PNI and CONUT. Vitamin D deficiency affects the regulation of progressive inflammation and metabolism in adipocytes and exacerbates the obesity process (26), which serves as an important pathological basis for its clinical indications. Previous studies have consistently revealed a correlation between vitamin D interventions and improvement in clinical outcomes. For example, a weekly therapeutic dose of 50,000 IU of vitamin D3 for at least 2 months could lower homocysteine levels in metabolomic syndrome, reducing the risk of cardiovascular disease in overweight women (27). Similarly, patients with diabetes with high serum vitamin D levels were found to have a reduced risk of all-cause mortality (28). Additionally, patients with cancer might benefit from vitamin D3 supplements to improve vitamin D status, potentially reducing cancer-related morbidity and mortality (29). Although the appropriate dose and method of supplementation for populations with different BMIs remain unclear (30), the association between vitamin D and readmission in this study is indicative of its clinical importance; thus, it is imperative to improve therapeutic strategies and reduce risk of readmission. Notably, studies have included perioperative complications as a key for clinical outcome (31), in addition to the predefined endpoint event of readmission here. Therefore, a comparison was further made between the baseline vitamin D levels of patients with and without perioperative complications, while no significant difference was found (*p* = 0.314). It was proposed that baseline vitamin D level was less associated with perioperative complications, but more with readmission in 1-year.

Particularly, the current study found a non-linear relationship between baseline 25(OH)D levels and postoperative readmission. The patterns of non-linearity appeared to be more generalized for 6-month readmission, showing a U-shaped association. As was shown in Figure 6, the risk of readmission decreased when the 25(OH)D level arrived at approximately 33 ng/mL and then increased as the levels turned higher or lower. The non-linear data from this study suggest that, in addition to overcoming vitamin D deficiency, the upper limit of supplementation should also be considered as a potential threat. Recommended target levels for vitamin D supplementation are differentiated by guidelines according to disease type, target population, and clinical outcomes, but most focus one-sidedly on the lower limit. Vitamin D toxicity may occur when serum 25(OH)D levels approach approximately > 150 ng/mL, with early manifestations of hypercalciuria and hypercalcemia (32). Existing data are insufficient to determine a proper upper limit for serum 25(OH)D. As reported, patients treated with high doses of vitamin D may have an increased risk of fractures, certain cancers, and even all-cause mortality (33, 34). In obese patients who has undergone gastrointestinal reconstruction, the oral dose of vitamin D and the duration of vitamin D treatment depends on the patients’ absorption levels. Daily supplementation with at least 2,000–4,000 IU of vitamin D was endorsed as recommended by serious of guidelines for post-bariatric patients (35), but there was no consensus on recommended plasma range. In line with previous studies, the trend in the right half of the RCS curve indicates an increased risk of readmission with incremental vitamin D supplements. For patients who continually go through vitamin D deficiency or insufficiency, treatment with more readily absorbed hydroxylated vitamin D metabolites or sunlight/sunlamps may be proposed (36). Thus, this study shows that the vitamin D classification criteria should be optimized for obese patients, and the prognostic effect should be considered in individual supplementation.

Furthermore, surgery can induce and exacerbate vitamin D reduction and antagonize the positive weight loss-inducing effects of bariatric surgery owing to post-operative physiological changes, weight loss, altered gut microbiome, loss of intrinsic factor/gastric acid, and medication use (37, 38). Risk of malabsorption is stratified in different surgery types. Malnutrition is more likely to ensue following the absorption-restricted gastric bypass surgery, rather than sleeve gastrectomy that bypasses the main absorption pathway (39). In this retrospective study, preoperative 25(OH)D levels (*p* = 0.375) and readmission rates (*p* = 0.956) were not statistically different between the two types of surgery. For the majority of enrolled patients undergoing sleeve gastrectomy (not applicable for gastric bypass), 25(OH)D was also identified as a prognostic indicator of 1-year readmission [OR 2.94 (95% CI, 1.03–8.39)], in addition to its overall significance. It was then suggested that although the malnutrition risk following sleeve gastrectomy was relatively low, vitamin D deficiency still turned to be a prognostic indicator in follow-up. Vitamin D deficiency is involved in the whole process from pre- to post-bariatric surgery. Even with regular oral supplementation, the serum levels could not improve effectively for several years (16). To improve postoperative vitamin D levels, studies have proposed prehabilitation to achieve preoperative weight loss and micronutrient deficiencies in the pre-operative period through lifestyle and other interventions, and ultimately optimize postoperative outcomes and reduce postoperative complications. In line with this, data from this study suggest that preoperative vitamin D is not only a prospective indicator of post-surgery outcome but also could be redeemed as an important intermediate indicator for the effectiveness of prehabilitation, providing new insights for clinical decision-making.

Seasonal variables were included in this study primarily to balance the potential influence on vitamin D levels (37, 40). Studies on the seasonal chronology of surgical complications regardless of the influence on 25(OH)D are limited. One preliminary study explored the seasonality pattern of perioperative adverse outcomes after bariatric surgery, denoting higher incidence of deep venous thrombosis and sepsis in colder seasons, and proposing the significance of seasonality (41). It is shown that the percentage of weight lost in the three months after bariatric surgery is higher during the summer months (July–November) (42). Meanwhile, seasonal factors regulate changes in ghrelin levels, with peaks in summer and autumn, resulting in increased appetite (43). Rapid weight loss and increased appetite will then increase the risk of gastrointestinal complications and postoperative infections. Above this, patient readmission may be influenced by a wide range of social factors, including cultural practices, lifestyle choices and work schedules (44).

This study has several limitations. Several patients were excluded because of missing important information, and preoperative supplement, an important covariate for baseline 25(OH)D levels, was also not recorded in this retrospective study, indicating less appreciation for vitamin D medication and monitoring in clinical routines. Even after a series of model modifications and robustness assessments, potential confounders and causal inferences could not be thoroughly assumed, adding to the variability of vitamin D levels. In addition, this study did not conduct detailed subgroup analysis due to the relatively homogeneous type of bariatric surgery and the low readmission rate during the 1-year follow-up. Furthermore, although this study provided new insights into the preoperative vitamin D evaluation and supplementation, its limitation as a retrospective study precluded it from providing dose-effect guidance for clinical practice. Vitamin D deficiency should be carefully managed in prospective cohort studies through individualized pharmacological interventions to reduce postoperative comorbidities.

In conclusion, this carefully matched retrospective cohort study demonstrates an increased risk of comorbidities in patients with low baseline vitamin D levels who undergo bariatric surgery in summer and autumn. The vitamin D level is an influential marker for assessing preoperative nutritional status. Importantly, serum 25(OH)D levels have a U-shaped relationship with 6-month readmission, and thus, they should be maintained within the desirable range through individualized medication to optimize patient outcomes.

## Data availability statement

The original contributions presented in this study are included in this article/Supplementary material, further inquiries can be directed to the corresponding author.

## Ethics statement

The studies involving humans were approved by the Ethics Committee of China-Japan Friendship Hospital. The studies were conducted in accordance with the local legislation and institutional requirements. The participants provided their written informed consent to participate in this study.

## Author contributions

YS: Conceptualization, Writing – original draft, Writing – review & editing. MC: Supervision, Writing – original draft, Writing – review & editing. TW: Supervision, Writing – original draft, Writing – review & editing. TX: Conceptualization, Data curation, Writing – original draft, Writing – review & editing.

## Supplementary material

Supplementary Figure 1 Sankey diagram to visualize and quantify PNI- and CONUT- defined malnutrition and 25(OH)D levels for readmitted patients (*n* = 25).
