# Supplementary figures and images for: Baseline 25(OH)D level is a prognostic indicator for bariatric surgery readmission: a matched retrospective cohort study
Source: Front Nutr. 2024 May 13;11:1362258. doi: 10.3389/fnut.2024.1362258 (PMC11128655; doi:10.3389/fnut.2024.1362258)

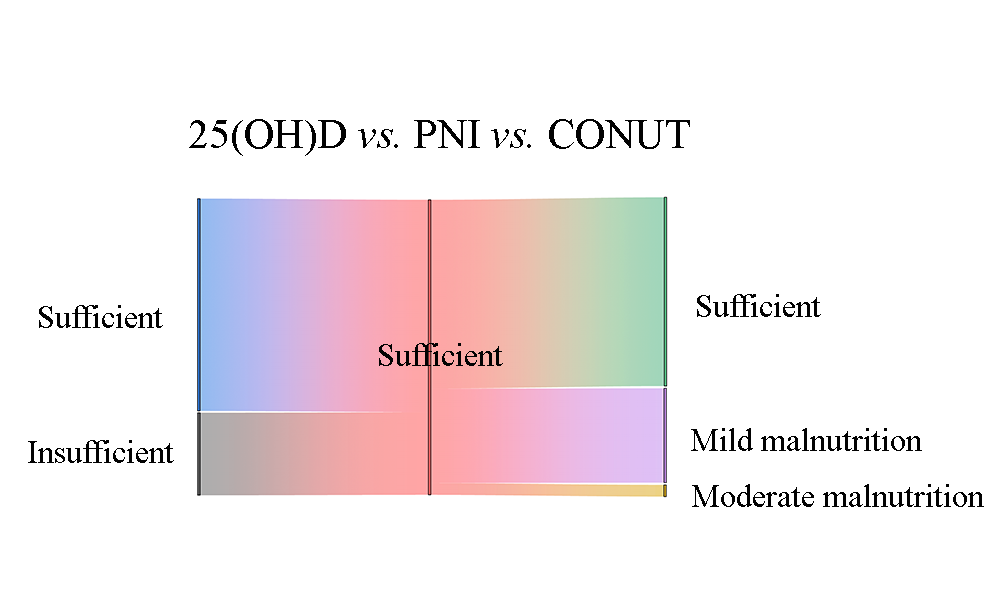

Supplement: Supplementary Figure 1 — Sankey diagram to visualize and quantify PNI- and CONUT- defined malnutrition and 25(OH)D levels for readmitted patients (n = 25). [file Image_1.tif]
